# Cardiovascular risk factors associated with frailty syndrome among hospitalized elderly people: a cross-sectional study: Fatores de risco cardiovasculares associados à síndrome de fragilidade em idosos hospitalizados: um estudo transversal

**DOI:** 10.1590/1516-3180.2016.0028010616

**Published:** 2016-09-26

**Authors:** Darlene Mara dos Santos Tavares, Camila Gigante Colamego, Maycon Sousa Pegorari, Pollyana Cristina dos Santos Ferreira, Flávia Aparecida Dias, Alisson Fernandes Bolina

**Affiliations:** I RN, PhD. Associate Professor, Department of Nursing Education and Postgraduate Program on Community Health Nursing, Universidade Federal do Triângulo Mineiro (UFTM), Uberaba, MG, Brazil.; II Undergraduate Student, Physiotherapy Course, Universidade Federal do Triangulo Mineiro (UFTM), Uberaba, MG, Brazil.; III MSc, PhD. Assistant Professor, Physiotherapy Course, Universidade Federal do Amapá (UNIFAP), Macapá, AP, Brazil.; IV RN, MSc. Doctoral Student, Postgraduate Course on Healthcare, Universidade Federal do Triângulo Mineiro (UFTM), Uberaba, MG, Brazil.; V RN, MSc. Doctoral Student, Postgraduate Course on Healthcare, Universidade Federal do Triângulo Mineiro (UFTM), Uberaba, MG, Brazil.; VI RN, MSc. Doctoral student, Ribeirão Preto School of Nursing, Universidade de São Paulo (USP), Ribeirão Preto, SP, Brazil.

**Keywords:** Frail elderly, Risk factors, Cardiovascular diseases, Aging, Hospitalization, Idoso fragilizado, Fatores de risco, Doenças cardiovasculares, Envelhecimento, Hospitalização

## Abstract

**CONTEXT AND OBJECTIVE:**

Identification of frailty syndrome and its relationship with cardiovascular risk factors among hospitalized elderly people is important, since this may contribute towards broadening of knowledge regarding this association within tertiary-level services. This study aimed to evaluate the cardiovascular risk factors associated with frailty syndrome among hospitalized elderly people.

**DESIGN AND SETTING:**

Observational cross-sectional study in a public teaching hospital.

**METHODS:**

The participants were elderly patients admitted to clinical and surgical wards. The cardiovascular risk factors assessed were: body mass index (BMI), waist circumference, systemic arterial hypertension (SAH), blood glucose, total cholesterol, high-density lipoproteins (HDL), low-density lipoproteins (LDL) and triglycerides. To identify frailty syndrome, the method proposed by Fried was used. The data were analyzed through descriptive statistics, chi-square test (P < 0.10) and multinomial logistic regression (P < 0.05).

**RESULTS:**

A total of 205 individuals were evaluated. It was found that 26.3% (n = 54) of the elderly people were frail, 51.7% (n = 106) were pre-frail and 22% (n = 45) were non-frail. The preliminary bivariate analysis (P < 0.10) for the regression model showed that frailty was associated with BMI (P = 0.016), LDL cholesterol (P = 0.028) and triglycerides (P = 0.093). However, in the final multivariate model, only overweight remained associated with the pre-frail condition (odds ratio, OR = 0.44; 95% confidence interval, CI = 0.20-0.98; P = 0.045).

**CONCLUSION:**

States of frailty were highly present in the hospital environment. The pre-frail condition was inversely associated with overweight.

## RESUMO

**CONTEXTO E OBJETIVO:**

A identificação da síndrome de fragilidade em idosos hospitalizados em interface com fatores de risco cardiovascular é relevante, pois pode contribuir para a ampliação do conhecimento sobre essa relação no nível de serviço terciário. Este estudo objetivou avaliar os fatores de risco cardiovascular associados à síndrome de fragilidade em idosos hospitalizados.

**TIPO DE ESTUDO E LOCAL:**

Estudo observacional transversal em hospital público de ensino.

**MÉTODOS:**

Participaram idosos internados nas clínicas médica e cirúrgica. Os fatores de risco cardiovascular avaliados foram: índice de massa corporal (IMC), circunferência abdominal, hipertensão arterial sistêmica (HAS), glicemia, colesterol total, lipoproteínas de alta densidade (HDL), lipoproteínas de baixa densidade (LDL) e triglicérides. Para identificar a síndrome de fragilidade, utilizou-se o método proposto por Fried. Os dados foram analisados por estatística descritiva, teste qui-quadrado (P < 0,10) e regressão logística multinomial (P < 0,05).

**RESULTADOS:**

Foram incluídos 205 indivíduos. Constatou-se que 26,3% (n = 54) dos idosos eram frágeis, 51,7% (n = 106) pré-frágeis e 22% (n = 45) não frágeis. A análise bivariada preliminar (P < 0,10) para o modelo de regressão indicou associações da fragilidade com as variáveis IMC (P = 0,016), colesterol LDL (P = 0,028) e triglicérides (P = 0,093). Entretanto, no modelo multivariado final, apenas a variável excesso de peso permaneceu associada à condição de pré-fragilidade (*odds ratio*, OR = 0,44; intervalo de confiança, IC 95% = 0,20-0,98; P = 0,045).

**CONCLUSÃO:**

Os estados de fragilidade apresentaram-se elevados em ambiente hospitalar. A condição de pré-fragilidade foi inversamente associada ao excesso de peso.

## INTRODUCTION

Frailty among elderly people can be understood as a clinical syndrome. It is characterized as a state of increased vulnerability to stressors that results from decreased physiological reserves and imbalances in multiple systems.^1^^,^^2^

Seeking to increase knowledge regarding this condition has become a worldwide investigative aim for researchers,^3^ since frailty may cause higher risk of health problems, hospitalization and mortality, as well as family overload and increased use of healthcare systems.^4^

A review indicated that cardiovascular diseases is a factor associated with frailty syndrome, since the prevalence of cardiovascular conditions may range from 25% to 50% among frail elderly people.^5^ Furthermore, the association between these conditions is bidirectional^6^ and low level chronic inflammation is present in both conditions.^5^

In a study involving community-living elderly people in England, it was found that the lowest frailty rate was among those with body mass index (BMI) 25-29.9 kg/m^2^. Elderly people with high waist circumference measurement were significantly more frail.^3^ Another population-based study in Great Britain reported higher odds of obesity, high waist circumference, low high-density lipoprotein-cholesterol (HDL) and hypertension among frail elderly men than among non-frail men.^7^

In Brazil, a study conducted in 17 cities found that BMI and abdominal circumference were associated with frailty, although obesity did not present any significant association.^8^ Additionally, in a study among community-living elderly people, frailty was associated with higher blood pressure, larger abdominal circumference and low blood HDL levels. Cardiovascular risk factors such as BMI, low-density lipoprotein (LDL) levels, total cholesterol and triglycerides were not associated with frailty.^9^ In a study carried out among hospitalized elderly people, BMI and arterial systemic hypertension were not associated with frailty syndrome.^10^

Thus, the findings from studies conducted in Brazil and in other countries are contradictory, which highlights the need for more studies in this area. Furthermore, most of the investigations have assessed community-living elderly people.^2^^,^^3^^,^^7^^,^^8^^,^^9^ There is a gap in the literature with regard to the hospital context, especially for the Brazilian elderly population.

Early identification of frailty syndrome among elderly people and correlation of its occurrence with cardiovascular risk factors may prevent disease progression and adverse outcomes.^11^ Within the hospital context, this knowledge can help in planning interventions for frail elderly people, with the aim of reducing the duration of hospitalization and occurrences of rehospitalization.

## OBJECTIVE

The aim of this study was to ascertain the cardiovascular risk factors associated with frailty syndrome among hospitalized elderly people.

## METHODS

This was an observational analytical cross-sectional study that formed part of a larger project entitled "Study of Frailty in Elderly People (EFRAGI)", funded by the National Council for Scientific and Technological Development (CNPq). The protocol was approved by the ethics committee of Universidade Federal do Triângulo Mineiro (UFTM) (document no. 2511). The interviewers approached the elderly individuals in the hospital, presented the study aims and explained the consent form, so as to make sure that there were no doubts regarding the research protocol, and then asked the patient to sign the consent form. Individuals were included when they were 60 years of age or over and were hospitalized in the clinical and surgical wards of a public teaching hospital in the city of Uberaba, located in the state of Minas Gerais, Brazil.

The sample size was calculated in accordance with the main objectives of the EFRAGI study. The prevalence of frailty was taken to be 30%, as observed in previous studies in hospital environments,^12^^,^^13^ with a precision of 5% and a confidence interval of 95% in a finite population of 1,455 elective elderly patients. The sample size required was found to be 265 elderly individuals. Moreover, a sample loss of 50% was taken into consideration and therefore the maximum number of interviews was 530. The recruitment process was carried out by means of systematic random sampling, with a sampling interval of 2. The elderly individuals were included in a chronologically organized list that took the time of hospitalization into consideration: the first individual was randomly chosen and the next was excluded; the third was included and the fourth was excluded; and the same pattern was followed until the end of the list.

The data collection encompassed the period from April 2013 to March 2014. The inclusion criteria were that the patients needed to be 60 years or over, without cognitive impairment, and needed to have agreed to participate in the study. Elderly individuals who presented restrictions on walking due to their recovery from surgery were interviewed on the next day. The exclusion criteria comprised the presence of severe cognitive impairment or Pfeffer scores greater than or equal to six, in association with absence of a caregiver; stroke sequelae with localized impairment of strength and aphasia; severe or unstable Parkinson disease associated with severe impairment of mobility, speech or illness that made it impossible to conduct the tests; end-of-life status, with severe impairment of vision and hearing; new hospitalization of an elderly individual who had already been included in the study; and presence of limitations regarding walking and talking.

Over the data collection period of the study, 445 patients were found to be eligible, but only 205 participated in the study because of losses and exclusions: refusal to participate (75), cognitive impairment without a caregiver (57), Pfeffer greater than or equal to more than six (44), absence of blood samples (50) and other reasons (14). Blood samples were collected on the day after data collection, by hospital laboratory staff. The patients who were excluded because no blood samples had been taken were discharged before the blood sample could be taken.

Before the beginning of the interview, cognitive impairment was assessed using the Mini-Mental State Examination (MMSE),^14^ which has been translated and validated for use in Brazil. This instrument is scored on a scale from zero to thirty points, and takes into consideration the elderly individual's schooling level: 13 points for illiterate individuals, 18 points for individuals with one to 11 years of schooling and 26 points for individuals with more than 11 years of schooling.^14^ In addition, if the elderly individuals presented cognitive impairment, as assessed through the MMSE, the Pfeffer questionnaire^15^ was filled out by the individual's caregiver. The Pfeffer questionnaire consists of an 11-question scale that is designed to evaluate elderly individuals' ability to perform some activities. The maximum score of this scale is 33 points, and it verifies the presence and severity of the cognitive impairment, considering functionality and the need for assistance from other people. In this study, the interview was conducted when the Pfeffer results were below six points, and supplementary information was requested from the caregiver, whenever necessary.

A structured questionnaire was used to obtain sociodemographic, economic and health data. The individuals who were able to walk underwent anthropometric evaluation consisting of measurements of weight, height and BMI. The patients who were unable to walk were assessed through estimated heights and weights using the equations proposed by Rabito et al.^16^ for assessing hospitalized individuals. The weight estimate calculation included arm circumference, abdominal circumference, calf circumference and arm semi-span.^16^

Nutritional status was classified using the BMI, and the cutoff points used were: underweight (BMI ≤ 22 kg/m²), normal weight (BMI between 22 and 27 kg/m²) and overweight (BMI > 27 kg/m²).^17^ In order to classify the patients based on abdominal circumference, the cutoff values of 102 cm and 88 cm, for men and women respectively, were used to consider whether an individual presented abdominal obesity.^18^

A blood sample was obtained within the first 24 hours of hospitalization, for a biochemical assessment. The patients' lipid profiles were interpreted in accordance with the Fifth Guidelines on Prevention of Dyslipidemia and Atherosclerosis, issued by the Brazilian Society of Cardiology.^19^ In order to consider that values were outside of their normal range, the following criteria were used: total cholesterol > 200 mg⁄dl; LDL (low-density lipoprotein cholesterol) > 100 mg/dl; HDL (high-density lipoprotein cholesterol) < 40 mg/dl; and triglycerides > 150 mg/dl. Fasting glycemia^20^ was considered altered when the value was above 100 mg/dl.

The presence of frailty syndrome was ascertained using a five-component phenotype proposed by Fried et al.,^1^ as follows:

1. Unintentional weight loss, assessed by means of the question "In the last year, did you unintentionally lose 4.5 kg or more (i.e. without diets or exercise)?"
2. Decreased muscle strength, verified through handgrip strength, which was assessed through three measurements presented in kilograms-force (kgf), with one-minute intervals between the measurements. The average value from the three measurements was taken and the cutoff point proposed by Fried et al.^1^ was used.
3. Self-reported exhaustion, assessed through two questions from the Brazilian version of the depression scale of the Center for Epidemiological Studies (CES-D):^21^ "Have you felt that you had to make an effort to complete your daily activities?" and "Were you unable to carry on with your things?" The participants were asked how they felt in the past week regarding these two questions, and the responses were obtained on a scale from 0 to 3, in which never or rarely was equal to 0; sometimes = 1; frequently = 2; and always = 3. Questions scored as two or three fulfilled this frailty criterion.
4. Walking slowness, which was assessed through the time taken to walk a 4.6 m distance, in seconds. The elderly individuals walked 8.6 m, and neither the initial nor the final two meters was taken into consideration in calculating the time taken. Furthermore, three measurements were made and the average value between them was used in the classification. This classification took into consideration the cutoff points proposed by Fried et al.^1^
5. Low physical activity level, which was ascertained through the long version of the International Physical Activity Questionnaire (IPAQ), as adapted for use in elderly populations.^22^ The classification adopted for this component followed the recommendations of the American College of Sports Medicine and the American Heart Association. These associations consider individuals to be "active" if they participate in more than 150 minutes of physical activities in a week, and "inactive" if they participate in zero to 149 minutes of physical activities in a week.^23^

The elderly individuals were considered to be frail when they presented three or more of the abovementioned components; pre-frail when they presented one or two components; and non-frail when they did not present any of the five components.^1^

The variables studied were:

1. social, demographic and economic: gender (male and female), age (60⎮-70; 70⎮-80; or 80 years and over), marital status (single, married/living with a partner, widower or divorced), number of years of schooling (none; 1⎮-4; 4⎮-8; 8; 9⎮-11; or 11 or more) and monthly income in minimal wages (no income; < 1; 1; 1-⎮3; 3-⎮5; or > 5);
2. self-reported morbidity: systemic arterial hypertension;
3. nutritional status, as assessed using the BMI: underweight, normal weight or overweight;
4. abdominal circumference in cm;
5. glycemic level in mg/dl;
6. total cholesterol in mg/dl;
7. HDL in mg/dl;
8. LDL in mg/dl;
9. triglycerides in mg/dl; and
10. frailty classification (non-frail, pre-frail and frail).

The data were input with double entry, to check for any inconsistencies. The stored data were then imported to the Statistical Package for the Social Sciences (SPSS) software, version 17.0, for analysis. 

The nominal variables were analyzed using absolute and percentage frequencies. Moreover, in order to identify the risk factors associated with the condition of pre-frailty or frailty, a preliminary bivariate analysis was carried out using the chi-square test, in which the results were considered significant when P < 0.10. Thus, the variables identified (P < 0.10) were included in a multivariate regression model. In addition, the factors that were associated with the pre-frail or frail conditions were identified by means of multivariate analysis using the prevalence odds ratio, which was ascertained through multinomial logistic regression (saturated model), taking a significance level of 5% and confidence interval of 95%. The predictors were: SAH, BMI, abdominal circumference, glycemia, total cholesterol, HDL, LDL and triglycerides.

## RESULTS

The prevalence of the non-frail condition among elderly individuals was 22% (n = 45). The prevalence of the pre-frail condition was 51.7% (n = 106) and the prevalence was 26.3% (n = 54) for non-frail individuals. Furthermore, there was a higher prevalence of frail female elderly individuals, while among non-frail and pre-frail individuals, there was higher prevalence of men. The marital status of widower accounted for 33.3% of the frail elderly people (Table 1). Our sample was mainly composed of individuals in the age category 60⎮-70 years who were married or living with a partner, with schooling level of 1⎮-4 years and with individual monthly income of one minimum wage (Table 1). The distribution of the social and demographic data for each frailty level is displayed in Table 1.

**Table 1:**
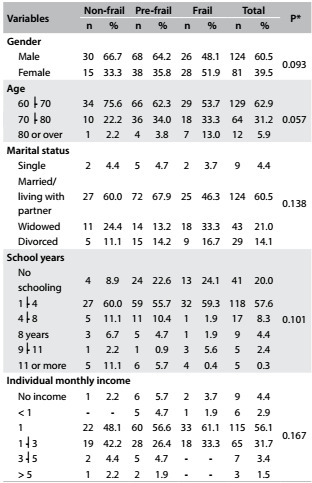
Distribution of social, demographic and economic variables in each frailty level among hospitalized elderly individuals. Uberaba, MG, Brazil, 2013

*Chi-square test.

Regarding the associated factors, the variables that were accepted in the preliminary bivariate analysis, in accordance with the inclusion criterion (P < 0.10), were: BMI (P = 0.016), LDL (P = 0.028) and triglycerides (P = 0.093) (Table 2). Therefore, these factors were included in the multivariate analysis.

**Table 2:**
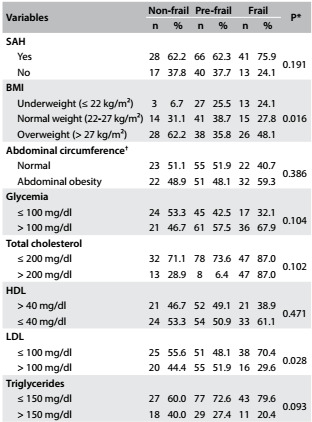
Cardiovascular risk factors associated with frailty level in hospitalized elderly individuals. Uberaba, MG, Brazil, 2013

*P < 0.10; ^†^Cutoff values of 102 cm and 88 cm, for men and women respectively, were used to consider that individuals presented abdominal obesity;^18^ SAH = systemic arterial hypertension, BMI = body mass index; HDL = high-density lipoprotein; LDL = low-density lipoprotein.

The variables relating to cardiovascular risk factors and their associations with frailty level are presented in Table 2.

In the multivariate model, overweight was inversely associated with the pre-frail condition (P = 0.045). Nevertheless, no other variables presented associations with frailty syndrome (Table 3). The variables included in the multivariate logistic regression (P < 0.05) are displayed in Table 3.

**Table 3:**
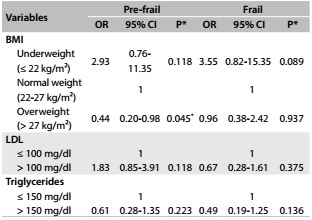
Final logistic multinomial regression model on the association between cardiovascular risk factors and pre-frail and frail conditions among hospitalized elderly individuals in Uberaba, MG, Brazil, 2013

OR = odds ratio; CI = confidence interval; 1: reference category; Non-frail: reference category; ^*^P < 0.05; BMI = body mass index; LDL = low-density lipoprotein.

## DISCUSSION

The prevalence of frail and pre-frail conditions among hospitalized elderly people differs between studies conducted in Brazil and elsewhere in the world.^10^^,^^24^^,^^25^ A study conducted in a Brazilian city found that the highest percentage (49.5%) of the elderly subjects were considered pre-frail,^10^ which corroborates the findings of the present study. Moreover, other investigations on hospitalized elderly people in Belgium^24^ and Norway^25^ obtained similar results, with values between 58.5% and 47.7%. However, the percentages of frail elderly individuals were higher in those investigations^10^^,^^24^ than in the present study (46.5%; 40%). These conflicting prevalences may be explained by differences in the inclusion criteria and in the diagnostic criteria for identifying frailty syndrome. Moreover, the age groups were also different, which were over 65 years^10^ and over 70 years.^24^ Additionally, the regional specificities of health status impairment among hospitalized elderly people may explain the differences between the findings.

The rate of occurrence of frailty syndrome may vary according to social, demographic and economic characteristics, as demonstrated in the descriptive data analysis. The predominance of frail women agrees with the findings from another Brazilian study^4^ conducted in a hospital, which showed that 63.6% of the frail elderly subjects were women. That study used the Edmonton Frail Scale (EFS) to make assessments. Moreover, in a recent literature review,^26^ female gender was positively associated with frailty. Therefore, this finding corroborates the hypothesis that women present more intense muscle mass loss due to ageing, since the changes in the estrogen levels after the menopause contribute towards this condition.^27^^,^^28^

Regarding marital status, the highest percentage of the frail elderly individuals consisted of widowers. This finding is supported by a Brazilian study^4^ conducted in hospital settings, in which 53.1% of the elderly subjects presenting severe frailty were single, divorced or widowers.

Among community-living elderly people, a similar result was found in an investigation in Taiwan, which found that 53% of the frail elderly individuals were widowers.^29^ Moreover, frailty syndrome involves complex interactions that include not only clinical factors but also social factors.^30^ Therefore, social support components such as widower status should be considered to be potential causal factors for the development of frailty syndrome.

The association between frailty syndrome and BMI, among individuals with low and high BMI, has been discussed in the literature.^3^ One investigation found that there was a strong positive association between BMI and frailty status at the baseline, in which participants who were overweight and obese were identified as presenting higher probability of becoming frail.^31^ However, in the present study, the condition of overweight was correlated as a protective factor against frailty, and this is supported by a review study that suggested that overweight was beneficial to elderly individuals, because of reduction of all-cause mortality.^32^ This may be important, given the repercussions of hospitalization and the intermediate condition of frailty that includes non-intentional weight loss. Nevertheless, some interventions directed towards elderly individuals within this setting might have influenced this result.

BMI has been shown to be an indicator for nutritional risk assessment and measurement of body fat, and is a diagnostic parameter for overweight and obesity. However, it presents some limitations, since it does not consider body composition and its distribution, which could lead to under or overestimation of body fat.^32^^,^^33^ Another point that should be taken into consideration is sarcopenic obesity, which consists of increased levels of adipose tissue relating to the ageing process, regardless of BMI, with higher deposition of visceral fat and muscle infiltration.^34^

Concerning the absence of association of LDL and triglycerides with the condition of pre-frailty, another Brazilian study^9^ also found similar results, in which LDL (P = 0.52) and triglycerides (P = 0.65) did not present differences. A prospective investigation in Finland^35^ found similar percentages, without significant differences (P = 0.45) in triglyceride and frailty levels. However, in a British prospective study, the triglyceride levels were higher among frail (12%) and pre-frail individuals (9%) than among non-frail individuals (7%), which disagrees with the results from the present study.^7^ In another study among elderly individuals, slight or moderate elevations in triglyceride and LDL levels were observed.^19^ Consequently, occurrences of lipid problems, among others, are risk factors for arterial coronary disease,^36^ which is related to frailty.^5^

The present study had some limitations:

1. the acute condition that led to hospitalization may have influenced both the diagnosis of frailty and the cardiovascular risk factors;
2. the group assessed was of limited size, given the small sample of individuals who met the inclusion criteria.

Nevertheless, the possibility of selection bias in the study was minimized, since only the subjects who met these criteria were included. Therefore, in view of these limitations, further research should be carried out with the aim of expanding the sample size and including morbidities as potential confounders.

## CONCLUSION

The prevalence of pre-frail status was 51.7%, while 26.3% of the elderly individuals studied presented a frail condition. Regarding the cardiovascular risk factors associated with frailty syndrome, only overweight remained significantly and inversely associated with pre-frailty status in the final model.
